# Reconstructing a Deblurred 3D Structure in a Turbid Medium from a Single Blurred 2D Image—For Near-Infrared Transillumination Imaging of a Human Body

**DOI:** 10.3390/s22155747

**Published:** 2022-08-01

**Authors:** Koichi Shimizu, Sihan Xian, Jiekai Guo

**Affiliations:** 1 Graduate School of Information, Production and Systems, Waseda University, Kitakyushu 808-0135, Japan; 2 School of Optoelectronic Engineering, Xidian University, Xi’an 710071, China

**Keywords:** transillumination, near infrared, scattering, turbid medium, 3D, reconstruction, image blur, PSF, deconvolution, focus stacking

## Abstract

To provide another modality for three-dimensional (3D) medical imaging, new techniques were developed to reconstruct a 3D structure in a turbid medium from a single blurred 2D image obtained using near-infrared transillumination imaging. One technique uses 1D information of a curvilinear absorber, or the intensity profile across the absorber image. Profiles in different conditions are calculated by convolution with the depth-dependent point spread function (PSF) of the transillumination image. In databanks, profiles are stored as lookup tables to connect the contrast and spread of the profile to the absorber depth. One-to-one correspondence from the contrast and spread to the absorber depth and thickness were newly found. Another technique uses 2D information of the transillumination image of a volumetric absorber. A blurred 2D image is deconvolved with the depth-dependent PSF, thereby producing many images with points of focus on different parts. The depth of the image part can be estimated by searching the deconvolved images for the image part in the best focus. To suppress difficulties of high-spatial-frequency noise, we applied a noise-robust focus stacking method. Experimentation verified the feasibility of the proposed techniques, and suggested their applicability to curvilinear and volumetric absorbers such as blood vessel networks and cancerous lesions in tissues.

## 1. Introduction

Important developments that have brought rapid progress to modern medicine include medical imaging techniques such as X-ray, MRI, and ultrasound imaging. Although they have been used widely in clinical practice, some difficulties remain. The methods, respectively, include difficulties of hazardous non-ionizing radiation, large-scale complicated equipment, and generally poor spatial resolution. Transillumination imaging with near-infrared (NIR) light is one candidate to resolve these difficulties for macroscopic imaging of the human body. It is safe for human health and presents many benefits in practical use [1,2,3,4,5].

NIR light at wavelengths of 700–1200 nm has relatively low absorption in light spectrum through body tissue. The light is detectable through a human head [6]. Some parts of internal structures of animal bodies can be visualized using NIR transillumination imaging (NIR-TI). In addition, functional imaging is possible to visualize the physiological functions inside the body using the principle of spectroscopy [7,8]. Because of these merits, NIR-TI has been used in clinical practice [8,9,10,11,12,13,14,15,16,17]. However, the image of a deep-seated structure is blurred severely because of strong light scattering in body tissues. The image only shows the structure close to the observed surface. The vein pattern obtained using the current NIR vein viewer is only a contrast-enhanced image of subcutaneous peripheral veins that are often visible to the naked eye with low contrast. These limitations of shallow imaging and two-dimensional (2D) imaging have hindered the widespread adoption of NIR-TI in medical fields that require useful macroscopic body imaging [4].

To overcome these limitations, NIR-TI must be advanced to 3D imaging. Depth estimation and tomography are the fundamental methodologies for 3D imaging. In recent decades, various attempts have been made for depth-resolved optical imaging through body tissue. They were summarized in reports of the relevant literature [1,2,3,4,5,18]. Among them, diffuse optical tomography (DOT) [19,20] and acousto-optic imaging techniques are applicable to macroscopic body imaging [21,22,23]. Although they are safe and useful, they require not only large computational effort and complicated instrumentation but also contact with the human body. NIR-TI does not have these constraints. Therefore, if we can solve the problem of scattering blur and attain 3D imaging, then NIR-TI can add another tool that is widely applicable to various medical fields.

As the basic principle for the solution, we noticed that the blur in NIR-TI includes information related to the depth of the light-absorbing structure in a turbid medium. Using this information, we can expect to estimate the depth of the absorber in the medium. Once we identify the depth, we can deblur the blurred image using the depth-dependent point spread function (PSF) in the process of reconstructing a 3D image. Based on this concept, we newly developed two techniques to estimate the absorber depth in a turbid medium. These techniques make it possible to reconstruct a deblurred 3D image from a single blurred 2D transillumination image. In the development, the simplicity in instrumentation was kept in mind for the technique to be used in the handheld device for bed-side point-of-care testing in clinical applications.

The first technique uses 1D information of the absorber image. The absorber depth is estimated using the look-up-table prepared beforehand. The second technique uses 2D information of the absorber image. A deblurred 3D image is reconstructed in the focus-stacking process. The first technique is simple and fast, but its effective use is limited to the absorber with curvilinear structure. The second technique is not as simple as the first, but it is applicable to a volumetric absorber and a curvilinear absorber. They are suitable for the NIR-TI of blood vessel networks and cancer/inflammation lesions, for example.

An attempt based on a similar concept has been made to reconstruct the 3D absorbing structure from a 2D transillumination image [24,25,26,27,28]. In these techniques, the absorber depth is estimated using the convolutional neural network obtained in deep learning processes. These techniques are effective for the same purpose, but they require great computational effort. They are unsuitable for real-time operation. The techniques proposed herein are much simpler and suitable for the primary clinical examination such as in emergency medicine. Although various other techniques are available to obtain the 3D image in a turbid medium [18,24,25,26,29,30], the methodology proposed herein is the first of its kind reported in the relevant literature.

## 2. Methods

### 2.1. NIR Transillumination Imaging

**Figure 1:**
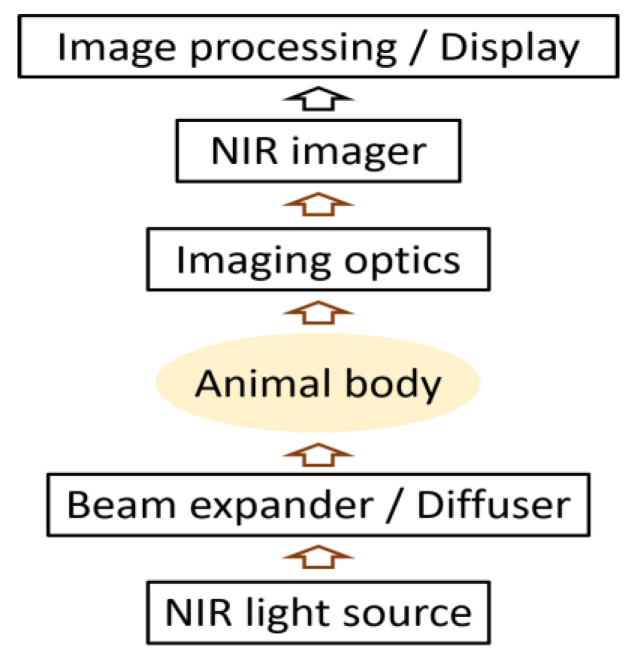
Concept of near-infrared transillumination imaging (NIR-TI).

**Figure 2:**
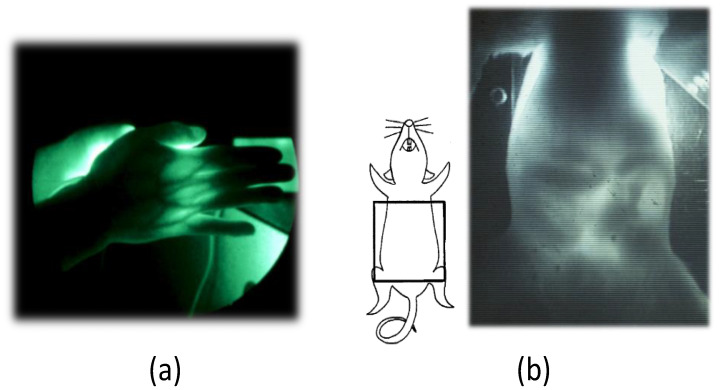
Examples of images obtained in NIR-TI: (**a**) human adult hand and (**b**) rat abdomen (one frame of video image).

Figure 1 depicts the NIR-TI concept for an animal body. A wide beam of light illuminates the body from one side. NIR light is widely spread in body tissue and becomes uniform back-projection lighting for the light-absorbing structure in the body. The transmitted light comes out from another side of the body forming the image through the body. Through some optical manipulation, the transmitted light is received by an imaging device. Then, image processing is performed to obtain the NIR transillumination image. Figure 2 presents examples of images obtained in NIR-TI [31,32,33]. Sufficient light transmission through body tissue is readily apparent when using the wavelength in the biological spectral window [1,2,3,4,5,6]. In Figure 2a, the peripheral vein network can be observed because of the large contrast of light absorption between the blood in veins and surrounding tissues.

Figure 2b depicts one frame of a video image showing the internal structure of a rat abdomen. In the video image, the movement of organs such as the peristaltic movement of intestines is visible in real time. It is noteworthy that the movement became visible with regular food intake with no artificial contrast agent. These images show common problems of NIR-TI. The deep-seated structure is severely blurred or is only slightly visible. The information is also limited in a 2D image.

### 2.2. Description of Blur in a Turbid Medium

The blur in NIR-TI can be described using a point spread function (PSF). The PSF for the turbid medium such as the body tissue was derived by the diffusion approximation of the energy transport equation [34] as shown below.

(1) $$PDF\left(\rho \right)=C\left[\left(\mu_{s}{}^{\prime }+\mu_{a}\right)+\left(\kappa_{d}+\frac{1}{\sqrt{\rho^{2}+d^{2}}}\right)\frac{d}{\sqrt{\rho^{2}+d^{2}}}\right]\frac{\exp\left(-\kappa_{d}\sqrt{\rho^{2}+d^{2}}\right)}{\sqrt{\rho^{2}+d^{2}}}$$

Therein, *κ*_d_ = [3*µ*_a_(*µ*_s_′ + *µ*_a_)]^1/2^, and the radial distance *ρ* = (*x*^2^ + *y*^2^)^1/2^. *C*, *µ*_s_′, *µ*_a_ and *d*, respectively, represent the constant with respect to *ρ* and *d*, the reduced scattering coefficient, the absorption coefficient, and the absorber depth.

**Figure 3:**
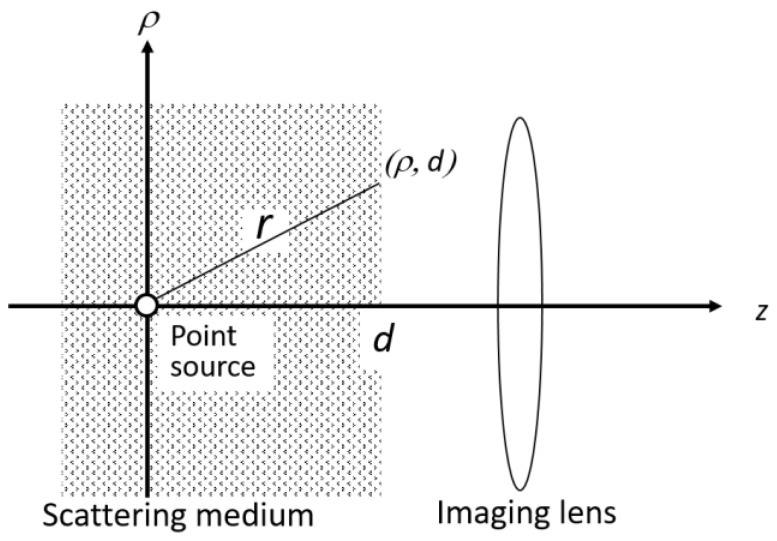
Theoretical model of depth-dependent point spread function (PSF).

Figure 3 portrays the theoretical model of the PSF. The PSF is the light distribution at the observation surface made with a point light source at depth *d*. The origin of the transillumination image is not a collection of light source points. It is rather a collection of shadow points. Nevertheless, these cases were demonstrated to be the same with sufficiently uniform illumination. The same PSF is useful for the shadow images [35]. The effectiveness and usefulness of this PSF were verified in simulations and experiments [34,35].

The proposed techniques in the following chapters require many blurred images in different conditions. With more data, better performance of the technique is expected. Using this PSF, it is not difficult to generate numerous blurred images. For example, one can generate as many blurred images as desired for the absorber with different shapes, sizes, and depths as

(2) $$I_{b}\left(x,y;d\right)=I_{o}\left(x,y\right)*PSF\left(x,y;d\right)$$

where *I_b_*(*x*,*y*;*d*), *I_o_*(*x*,*y*), *, and *PSF*(*x*,*y*;*d*), respectively, denote the blurred image, the original image without blur, the convolution operation, and the depth-dependent PSF.

### 2.3. Deconvolution with Depth-Dependent PSF

By reversing the process presented above, we can deblur the blurred image using PSF deconvolution as

(3) $$I_{o}\left(x,y\right)=I_{b}\left(x,y;d\right)⊛PSF\left(x,y;d\right)\;$$

where ⊛ signifies the deconvolution operation.

Therefore, if the depth of an absorber in a turbid medium is known, then the blurred image can be deblurred appropriately. The PSF deconvolution effectiveness for deblurring the transillumination images has been demonstrated in animal experiments [34,35].

However, the absorber depth is often unknown in medical applications. Obtaining a clear image of all parts of the absorber requires spatial distribution of the depth or 3D information about the absorber. Stereoscopic imaging techniques are used to obtain the 3D information in clear air [36], but they are generally not practical in a turbid medium such as body tissue. Confocal mapping techniques are another measure. They are useful in less-scattering media such as those used for cytological or histological analyses under a microscope [37]. However, it is difficult to focus NIR light in body tissues for macroscopic organ imaging in body tissues. Therefore, we devised new techniques to estimate the absorber depth using image blur as a depth index.

## 3. Depth Estimation with 1D Absorption Profiles

### 3.1. Principle of 3D Imaging

In NIR-TI of animal body, the image of interest often includes a curvilinear structure such as the network pattern of blood vessels. Figure 2a shows that the blood vessels appear as a dark curved thick line on the background of surrounding tissue. With deeper vessels, the image appears more blurred. Therefore, if we can quantify the blur appropriately, then we can expect to estimate the depth of the blood vessel. The typical indices to quantify the blur include the image intensity or brightness value, image contrast, image sharpness, edge profile or edge spread in the image. Considering the sensitivity and specificity in practical use in NIR-TI, we chose the image contrast and the edge spread as indices for depth estimation. They are parameterized as the Michelson contrast (*C_M_*) and the full width of half maximum (*FWHM*) of the intensity profile of an absorber image.

(4) $$C_{M}=\frac{I_{\max}-I_{\min}}{I_{\max}+I_{\min}}$$

Therein, *I*_max_ and *I*_min_, respectively, denote the maximum and minimum intensity values in the image.

Curvilinear absorber images such as blood vessels are divided into small pieces as fine as to be regarded as straight lines. Then, the intensity profile is obtained in the direction perpendicular to the axis of each piece of a linear absorber. The indices *C_M_* and *FWHM* are sensitive to the noise in the intensity profile. However, because the blood vessel image in NIR-TI is blurred severely, the major noise in the image is fundamentally random noise from the image-capturing device. Consequently, the simple low-pass filtering and the Gaussian fit can eliminate noise effects in obtaining these indices from the intensity profile. In this way, the depths of all small pieces of the absorber in the transillumination image can be estimated.

The depth estimation process is the lookup table method. We prepared the database for the lookup table, which connects a combination of *C_M_* and *FWHM* to the absorber depth. Once this table is ready, the depth of each part of the absorber can be estimated easily and quickly. Using this depth information, the image part can be deblurred by deconvolution with the depth-dependent PSF as Equations (1) and (3). Because of the simplicity of this process, it can be implemented in a straight stream of computer software from image capture to 3D image reconstruction. It can output clear 3D image in NIR-TI without much computational load. Real-time operation can be achieved using common technology.

### 3.2. Feasibility Check in Simulation

The feasibility of the proposed technique was examined through simulation. To produce the lookup table, many blurred images must be obtained in different absorber conditions such as depth, size, shapes and orientations. Although time consuming, generating the blurred images using Equations (1) and (2) was not difficult. Using the generated blurred images, we measured the *C_M_* and the *FWHM* of each profile and constructed the lookup table to connect the combination of *C_M_* and *FWHM* to the absorber depth.

**Figure 4:**
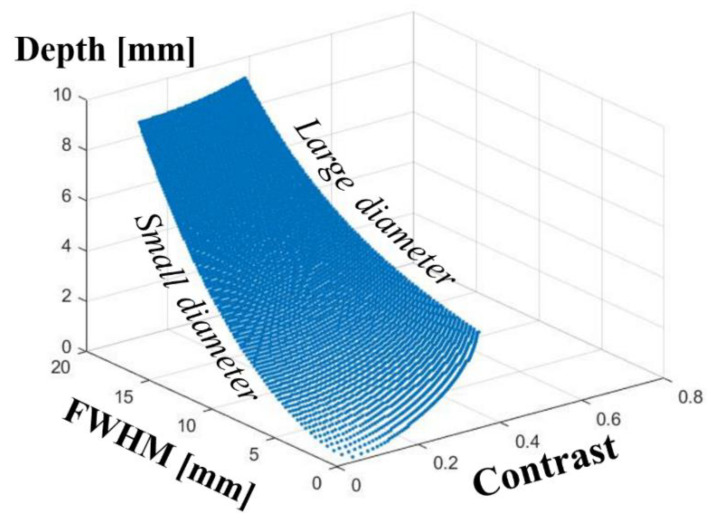
Example of lookup table to provide absorber depth and absorber thickness from the combination of contrast and spread of absorber intensity profile.

An example of the lookup table is portrayed in Figure 4. This table was made for the absorber diameter and absorber depth both from 1.0 to 10 mm with 0.1 mm increments. Good specificity or clear one-to-one correspondence is shown between the absorber depth and the combination of *C_M_* and *FWHM*. In Figure 4, the curved surface is a collection of curved lines. Each line corresponds to a certain absorber thickness. The lines in the left and right sides of the surface, respectively, correspond to the small and large thicknesses of a cylindrical absorber. No cross-talk was found to exist between the absorber depth and the absorber thickness in terms of estimation from the combination of *C_M_* and *FWHM* in this range. This finding suggests that we need not ascertain the absorber thickness a priori. In other words, the absorber thickness can be estimated from the combination of *C_M_* and *FWHM* simultaneously in the process of depth estimation.

**Figure 5:**
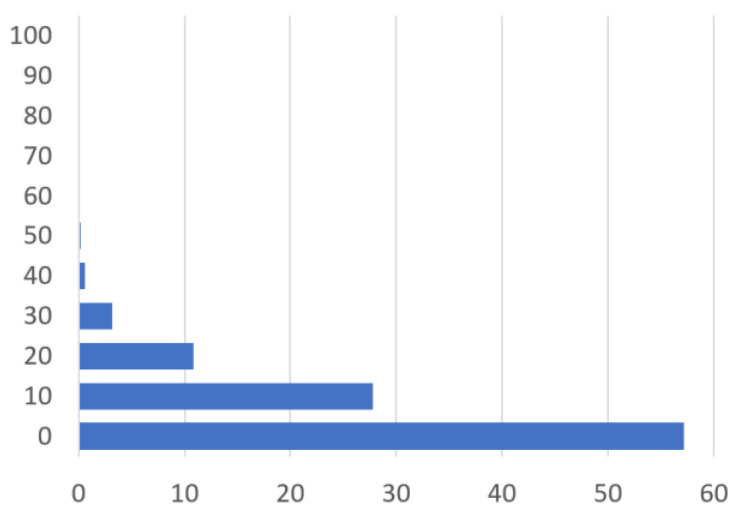
Estimation error statistics.

To test depth estimation accuracy, we generated blurred images using Equations (1) and (2). The conditions of test images were the following: an absorber diameter from 1.0 to 10 mm with 0.5 mm increments and an absorber depth from 0.1 to 10 mm with 0.01 mm increments. The same images used for the lookup table construction were eliminated from test images. The measured *C_M_* and *FWHM* values do not necessarily fall on the grid points of the lookup table. The estimated depth values were calculated from linear interpolation of the four depths obtained from the closest four grids of *C_M_* and *FWHM* combinations in the lookup table. Figure 5 presents error statistics in depth estimation. With 18,829 profiles, the inestimable cases were fewer than 0.14%. The average error was 6.3 μm. This result suggests that the proposed technique is effective to estimate the absorber depth from 0.1 to 10 mm in body tissues with submillimeter resolution and with an average error of micrometer order.

**Figure 6:**
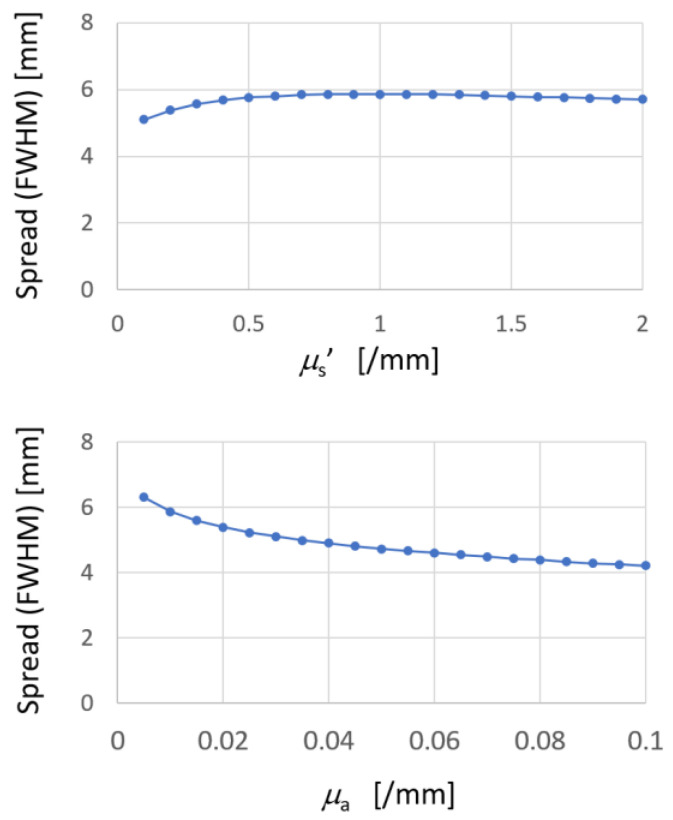
Dependence of PSF spread on variation in optical parameters.

During practical use of this technique with a living animal body, a change in optical properties of body tissues might occur. They are typically characterized by parameters of the reduced scattering coefficient (*µ*_s_′) and the absorption coefficient (*µ*_a_) of the turbid medium. If slight variation in *µ*_s_′ and/or *µ*_a_ in the physiological range produces a marked change in *C_M_* and *FWHM*, then estimation of the proposed technique becomes unstable, rendering its applicability as poor. To examine this effect, the dependence of PSF on these parameters in a practical range was investigated. Figure 6 presents examples of the dependence. The change in the width of PSF was much less than the change in *µ*_s_′ and *µ*_a_, indicating stability of the proposed technique against variation in the optical parameters of body tissues.

### 3.3. Applicability Test in Experiment

**Figure 7:**
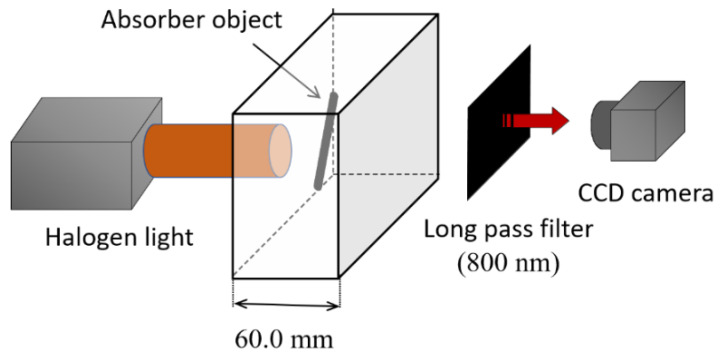
Experiment setup for NIR-TI.

After verification of the feasibility, we tested the applicability of the proposed technique using experimentation. Figure 7 shows the outline of the experiment schematically. A wide beam of light from a halogen lamp (0.4 mW/cm^2^/5 nm, PIS-UHX TWIN250; Nippon P-I) illuminated the turbid medium sample. The sample was contained in a rectangular parallelepiped plexiglass container with inner dimensions of 60 mm height, 100 mm width, and 60 mm depth in the direction of the light beam. The turbid medium was prepared by mixing a lipid emulsion (Intralipos 20%; Otsuka Pharmaceutical Co. Ltd., Tokyo, Japan) [38], black ink (Ink-350-B; Pilot Corp., Tokyo, Japan), and purified water. The optical property of the sample was controlled by their concentrations to simulate human body tissues, i.e., *µ*_s_′ = 1.0/mm and *µ*_a_ = 0.01/mm [1]. Transillumination images were captured using a CCD camera (30% QE at 800 nm, 1344 × 1024 pixels, C10600-10B; Hamamatsu Photonics K.K., Hamamatsu, Japan) through an optical filter (long-pass at 800 nm, ITF-50S-80IR; Sigma Koki Co., Ltd., Tokyo, Japan). With this filter, we could eliminate the stray light from room lighting with fluorescent lamps. The quantum efficiency of the CCD camera was less than 10% in the longer wavelength than 900 nm. In the turbid medium, we placed a light absorber, or a cylindrical metal bar painted in matte black with 3.0 mm diameter and 30 mm length. It was fixed at an inclination of 5 degrees from the vertical. The depths of the top and the bottom of the bar were 2.2 and 5.0 mm, respectively, as measured from the internal surface of the observation side. The steep angle was chosen for better depth estimation analysis than with the large angles from the vertical.

**Figure 8:**
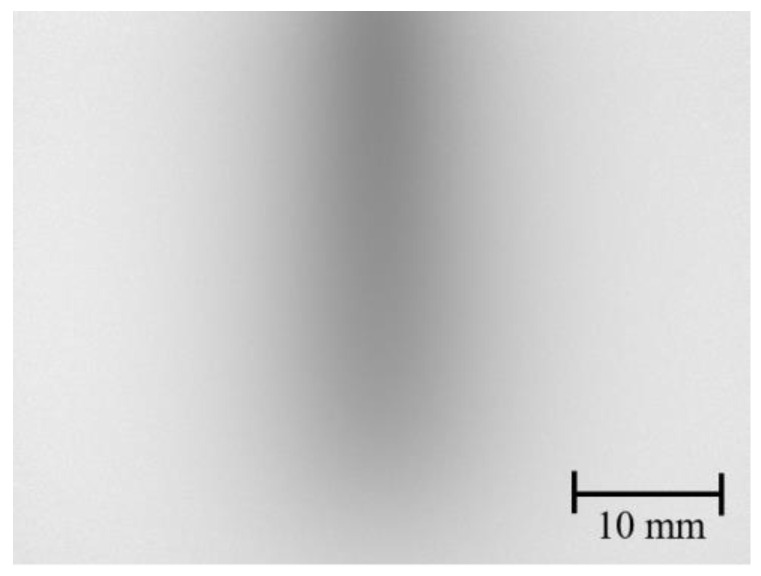
Example of transillumination image of inclined bar in a turbid medium.

**Figure 9:**
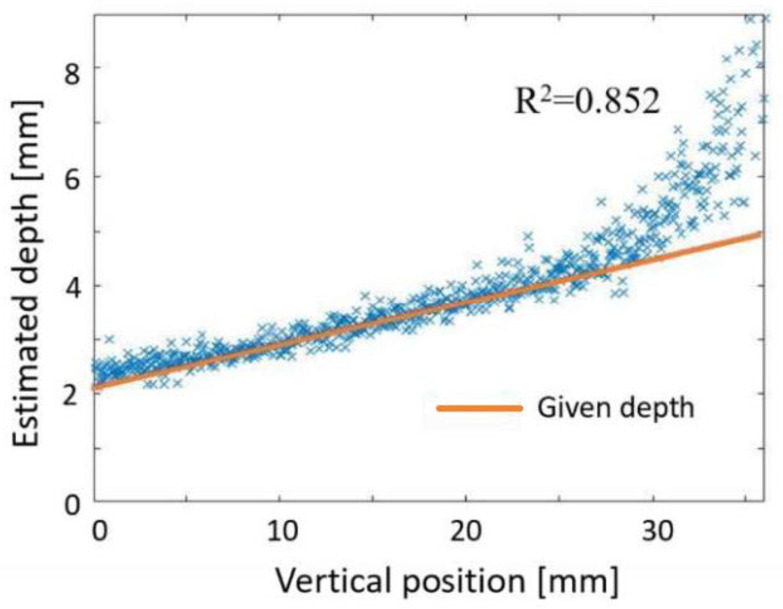
Results of depth estimation using 1D information of absorber image.

Figure 8 presents an example of the transillumination image of the inclined bar in the tissue-simulated turbid medium. The degree of image blur changes according to the absorber depth. Using the lookup table, the absorber depth can be estimated from the *C_M_* and the *FWHM* of the profile of the absorber image. The vertical length of this image consists of 720 pixels: 720 horizontal profiles were obtained. Figure 9 presents results of depth estimation obtained at each vertical position. We were able to estimate the correct depth as deep as 4.0 mm. Parts deeper than 4.0 mm were overestimated because the signal-to-noise ratio (SNR) of a transillumination image was insufficient. Although we tried to keep the measurement SNR 0.8–21 dB, the blur of the transillumination image was very severe in the deeper part. *C_M_* and *FWHM*, respectively, appeared as lower and larger than the correct values.

**Figure 10:**
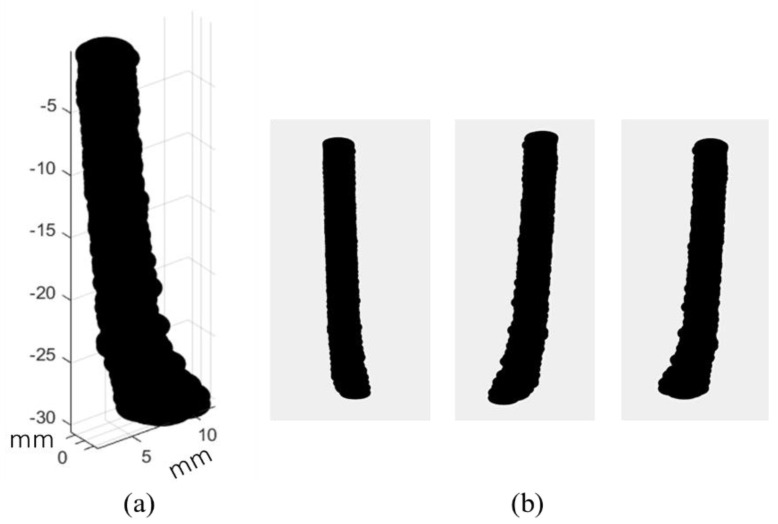
Results of 3D reconstruction using 1D information of absorber image: (**a**) reconstructed 3D image and (**b**) appearance from different view angles: the absorber was a right circular cylinder with smooth surface painted matte black, 3 mm in diameter and 30 mm in length. It was tilted 5 degrees from the vertical.

Using this depth information, we can deblur the blurred image by deconvolution with Equation (3). In addition, the lookup table provides information about the absorber thickness and depth. Therefore, if the cross-sectional shape of the absorber is known, then the 3D image of the absorber can be reconstructed using the estimated depth and thickness. In this experiment, we knew the absorber cross-section was a circle beforehand, and we were able to reconstruct a clear 3D image using the lookup table. The spatial resolution of the reconstructed 3D image is basically that of the depth estimation, or submillimeter in this case. Figure 10 presents results of this 3D reconstruction. Except for the part deeper than 4.0 mm, a 3D structure was reconstructed appropriately. This result demonstrates the applicability of the proposed technique to reconstruct a clear 3D structure from a single blurred 2D image obtained in NIR-TI.

## 4. Depth Estimation from 2D Image Information

### 4.1. Principle of 3D Imaging

The technique described in the preceding chapter is useful for clear 3D imaging of the binary curvilinear structure such as the blood vessel network contrasted against the background of surrounding tissue. Nevertheless, it is unsuitable for widely distributed structures such as the spread of cancerous tissue or inflammation lesions. We developed another technique to reconstruct the 3D structure of a clear image from a single blurred 2D image obtained in NIR-TI. This technique is based on the principle of focus stacking, also called focal plane merging, z-stacking or focus blending [39].

A transillumination image consists of parts with different blurring that correspond to different depths of the parts in a turbid medium. If we deblur the 2D image using the depth-dependent function with specific depth *d*, the image part of depth *d* was deblurred best, whereas other parts with different depths remain blurred. Therefore, by selecting the parts in focus, we can estimate the part depth and deblur the image of the part simultaneously. By repeating this process with varying depths *d*, we can reconstruct the 3D structure of the absorber in the turbid medium.

To realize this idea, we developed a technique using the PSF of Equation (1) as the depth-dependent function. A blurred transillumination image *I_b_*(*x*,*y*) is deconvolved with the depth-dependent point spread function *PSF*(*x*,*y*;*d*) producing many deblurred images *I_db_*(*x*,*y*;*d*) for different depths *d*, or

(5) $$I_{db}\left(x,y;d\right)=I_{b}\left(x,y\right)⊛PSF\left(x,y;d\right)$$

By specifying *m* depths, we can produce *m* deblurred images, or *I_db_*(*x*,*y*;*d_i_*), *i* = 1, …, *m*. Among the *m* images, we find the best focus image in each image part with the depth used for deconvolution. As a focus criterion, the gray-level variance of the image part was used. Both the PSF deconvolution and the selection using the gray-level variance are simple to calculate and are useful for blurred transillumination images. However, the following difficulties hinder their practical use. In deconvolution with PSF, the image component of high spatial frequency tends to be enhanced. The granular background noise or scratch-like noise becomes more noticeable than before the deconvolution. This makes accurate depth estimation difficult and degrades the reconstructed image. Therefore, we used a noise-robust selective fusion technique [40] to overcome these difficulties in depth estimation and 3D reconstruction.

### 4.2. Process Used to Estimate the Depth Distribution

The depth of the absorber in a turbid medium is estimated using the following steps.

(1) Deconvolution of a blurred image:

Multiple deblurred images *I_db_*(*x*,*y*;*d_i_*), *i* = 1, 2…m, are obtained from Equation (5) using the depth-dependent PSF given in Equation (1).

(2) Extraction of work area:

In the deblurred image, we sample the image part *i*_db_(*x*,*y*;*d_i_*) using the rectangular window with *r* × *r* pixels. With a larger *r*, we can suppress the effect of the high-frequency noise better. However, the spatial resolution and the selectivity of the best focus become poor with the large *r*. In this trade-off, we set *r* = 9 for our study [40].

*i_db_*(*x*,*y*;*d_i_*) = *I_db_*(*x*,*y*;*d_i_*) [*u*(*x*) − *u*(*x* − *r*)] [*u*(*y*) − *u*(*y* − *r*)] (6)

In that equation, *u*(*x*) is a unit step function.

(3) Focus criterion: To ascertain the best focus, we use the gray-level variance as a focus criterion and define it as focus function *fc*(*d_i_*), or

(7) $$fc(d_{i})=\overset{r\times r}{\underset{k=1}{\sum }}{\left[i_{k}\left(x,y;d_{i}\right)-\bar{i_{r}}\left(d_{i}\right)\right]}^{2}$$

where $\bar{i_{r}}$ is the mean intensity in window, or $\bar{i_{r}}\left(d_{i}\right)=\frac{1}{r\times r}\overset{r\times r}{\underset{k=1}{\sum }}i_{k}\left(x,y;d_{i}\right)$.

(4) Gaussian fit:

Focus function *fc*(*d_i_*) is fitted to a Gaussian function *g*(*d_i_*). The best focus depth *d*_max_ was estimated as the depth at which *g*(*d_i_*) has its peak. In this way, the estimation error caused by the high-spatial-frequency component in the image such as the random noise is suppressed effectively.

(5) Processes (2)–(4) are repeated with the work area scanned by shifting the rectangular window over the whole transillumination image.

### 4.3. Noise Suppression in 3D Reconstruction

In the process described earlier, we can obtain an image in which all parts are in the best focus. Using the estimated depth distribution, we are able to reconstruct a 3D image. However, the image quality is poor because high-spatial-frequency noise such as the granular background caused by the random noise from image capturing processes was enhanced in the deconvolution process. To suppress this noise while maintaining a clear image of interest, we followed the noise-robust selective fusion technique [40].

In deconvolved images *I_db_*(*x*,*y*;*d_i_*) with different depths *d_i_*, a consistent image-noise appears in the narrower range of *d_i_* than the major structure of interest. In contrast, random noise tends to appear in a wider range of *d_i_*. Therefore, summation of the deblurred image with appropriate weight function *w*(*d_i_*) can suppress its effects on the final image quality, or

(8) $$\hat{i}\left(x,y\right)=\overset{n}{\underset{i=1}{\sum }}w(d_{i})\;i_{i}\left(x,y;d_{i}\right)/\overset{n}{\underset{i=1}{\sum }}w(d_{i})\;$$

where *n* is the number of the weighted sum covering all the images to be summed up.

For the weight function *w*(*d_i_*), the normalized focus function $\bar{fc}\left(d_{i}\right)$ can be used.

(9) $$\bar{fc}\left(d_{i}\right)=fc\left(d_{i}\right)/\overset{n}{\underset{i=1}{\sum }}\;fc\left(d_{i}\right)$$

However, Equation (8) with the normalized focus function works as a low-pass filter (LPF) to degrade the sharpness of the main structure of the image. Weight function *w*(*d_i_*) should have a peak value at the focus depth *d*_max_. In image parts that include the main structure of interest with a high signal-to-noise ratio (SNR), the peak should be as narrow as possible to avoid the LPF effect. In contrast, in noise-dominant image parts, weight function *w*(*d_i_*) should be as wide as possible to suppress high-spatial-frequency noise. To address these contradictory demands, we used the following weight function [40].

(10) $$w\left(d_{i}\right)=\left\{1+\tanh\left[\phi \left(\bar{fc}\left(d_{i}\right)-1\right)\right]\right\}/2$$

Therein, the weight function sharpness is controlled by the sharpening parameter ϕ(s) of a function of the selectivity *s*(*x*,*y*;*d_i_*) or the peak SNR given as shown below.

(11) $$\phi \left(s\right)=\left\{1+\tanh\left[\alpha \left(s-s_{th}\right)\right]\right\}/\left(2\alpha \right)$$

(12) $$s\left(x,y;d_{i}\right)=20\log_{10}\{\max[fc\;(d_{i})]/\mathrm{RMS}[fc(d_{i})-g(d_{i})]\}$$

In that equation, *s_th_* stands for the threshold of the selectivity. *α* is the selectivity constant. *s_th_* specifies the SNR threshold to ascertain whether the image part is the main structure or noise. Additionally, *α* controls the strength of the selectivity. max and RMS signify the maximum and the root mean square operations.

### 4.4. Feasibility Check for Depth Estimation

The feasibility of the proposed technique was examined by experimentation. The experimental setup is fundamentally that shown in Figure 7. The light source was replaced by an array of 50 LEDs (QBHP684-IR3AU; QT-Brightek Corp., Milpitas, CA, USA) to improve the uniformity of the illumination for the 2D information processing. The optical power of each LED and the wavelength were 250 mW and 850 nm, respectively. The absorber in the turbid medium is a metal cylinder of 2 mm diameter painted matte black. Other conditions were the same as those shown for Figure 7.

**Figure 11:**
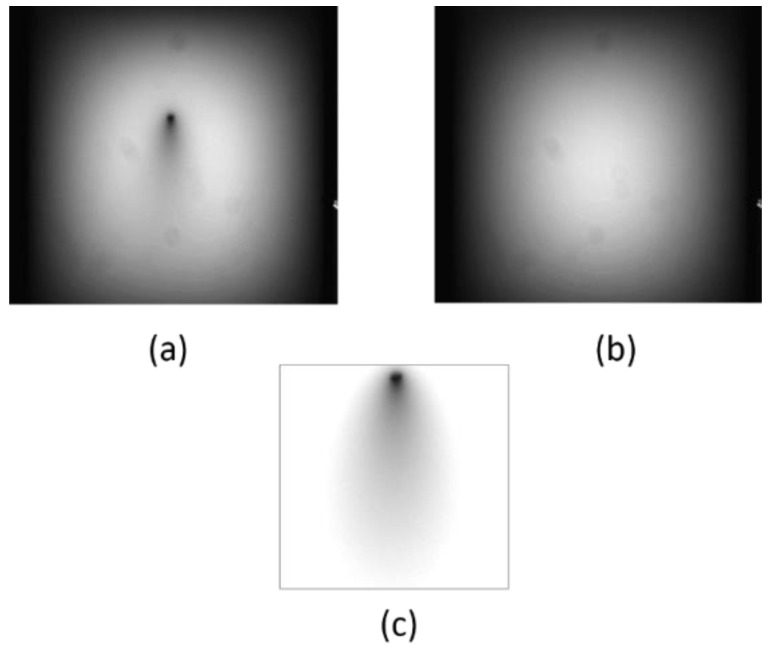
Background removal in transillumination image: (**a**) image with absorber, (**b**) image without absorber and (**c**) result of background removal.

**Figure 12:**
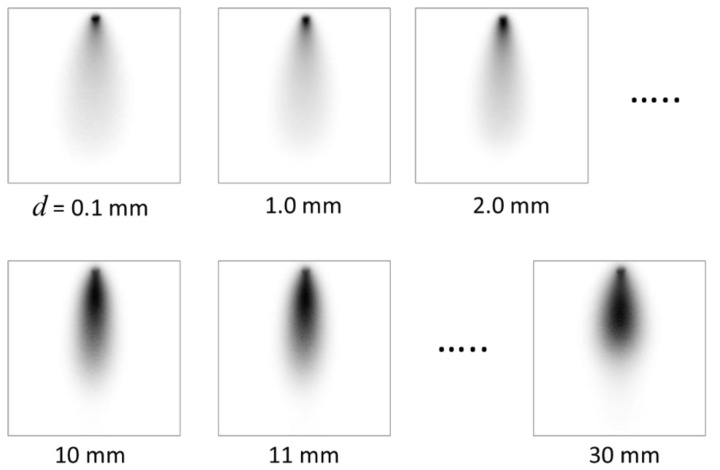
Examples of deconvoluted images with different depths.

**Figure 13:**
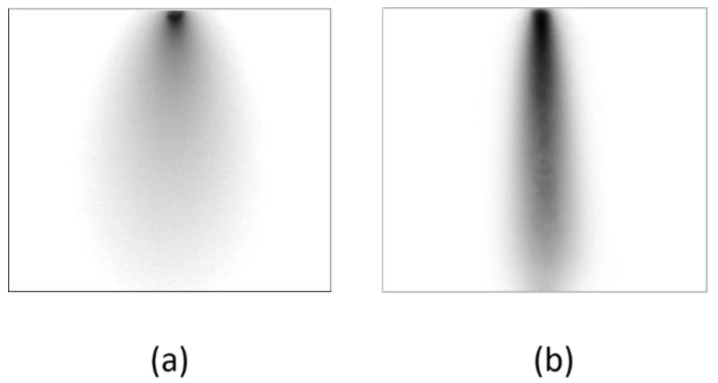
Results of focus stacking: (**a**) original blurred image and (**b**) after focus stacking.

To assess depth estimation accuracy, we placed the absorber in an inclined position in the turbid medium. We made the optical parameters of the medium to simulate the body tissue, or *µ*_s_′ = 1.0/mm and *µ*_a_ = 0.01/mm [1]. The depth of the bar, as measured from the inner surface of the container, was 0–30 mm. The background inhomogeneity was removed by dividing the intensity distribution of the image with the absorber by that without the absorber. Figure 11 shows the transillumination images with an absorber (Figure 11a), without an absorber (Figure 11b) and after background removal (Figure 11c). The image after background removal is deconvolved with the depth-dependent PSF using Equations (1) and (5). Depth *d* was varied from 0.1 mm to 1.0–30 mm with 1 mm increments. In this process, 31 deconvolved images with different focus depths were produced. Figure 12 portrays some examples of these images with the depth of the PSF used for the deconvolution. The process described in Section 4.3 was applied. The focus-stacking result is presented in Figure 13 together with the original image obtained in NIR-TI.

**Figure 14:**
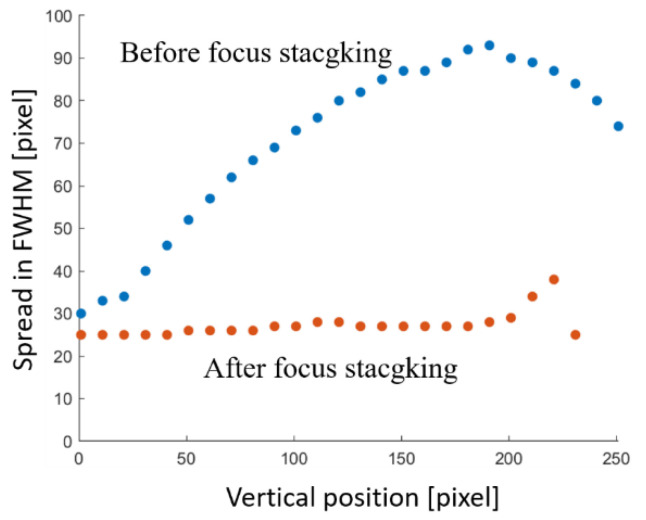
Effect of blur suppression by focus stacking.

For quantitative analysis, the intensity profile of the image in horizontal direction was obtained from the top to the bottom of the image. The image blur is measured in the FWHM of each profile. Figure 14 presents the result. Using the proposed technique, the absorber image blur was suppressed effectively; from a 110% to a 28% increase at 30 mm depth.

**Figure 15:**
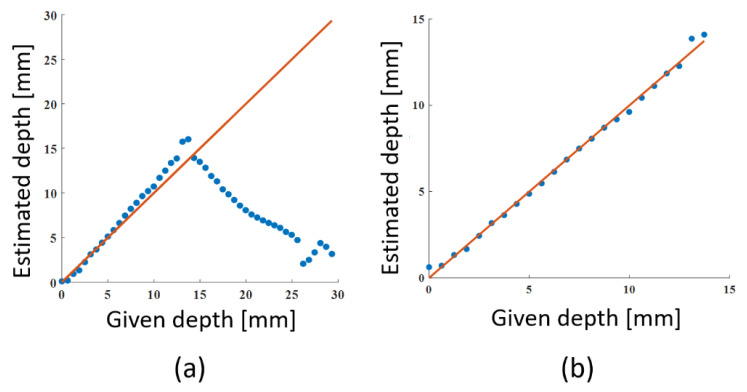
Results of depth estimation: (**a**) estimated depth in focus stacking, and (**b**) depth estimation using the calibration curve. Dots and a line show estimated and given depths, respectively.

Figure 15a presents a comparison between the given depth and the estimated depth. At the depth range of 0–13.5 mm, the estimated depth showed a monotonic increase with the given depth, but it turned to a decrease at depths greater than 13.5 mm. Although we tried to keep the measurement SNR 0.8–21 dB, the blur of the transillumination image was very severe in the deeper part. The low SNR made the image calculation systematically erroneous. However, as long as the results were within the available range, the maximum estimation error was 15%. Because the correlation between the given and estimated depth was very high (*R*^2^ > 0.99), we can calibrate the data for simple linear regression of the first order. Figure 15b presents the relation after calibration. The estimation error was reduced to less than 7.4% at depths of 0–13.5 mm. This result also suggested the submillimeter depth resolution of the proposed technique.

### 4.5. Verification of 3D Reconstruction by Experimentation

**Figure 16:**
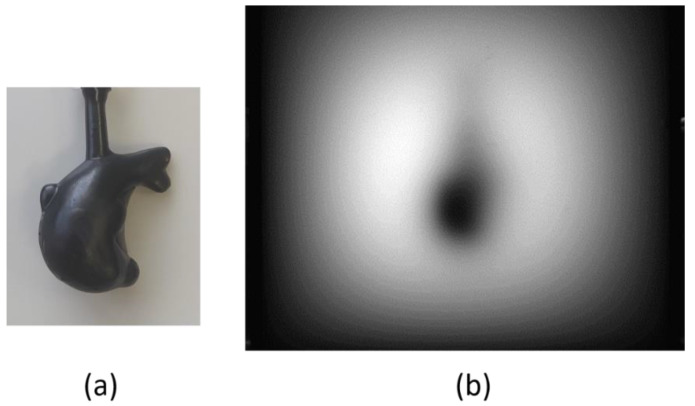
Appearance of a volumetric absorber: (**a**) appearance in air, and (**b**) image in NIR-TI.

One purpose of the proposed technique is 3D imaging of an absorbing structure in a turbid medium such as a subcutaneous cancerous lesion. To assess the applicability of the proposed technique, 3D reconstruction was attempted using a volumetric absorber with irregular shape. The measurement conditions were the same as those described in the preceding section (Section 4.4), except for the absorbing object. The absorber was a key-chain charm of a fat dolphin shape painted in matte black. Figure 16 shows its appearance in air and the image taken in the turbid medium in NIR-TI.

**Figure 17:**
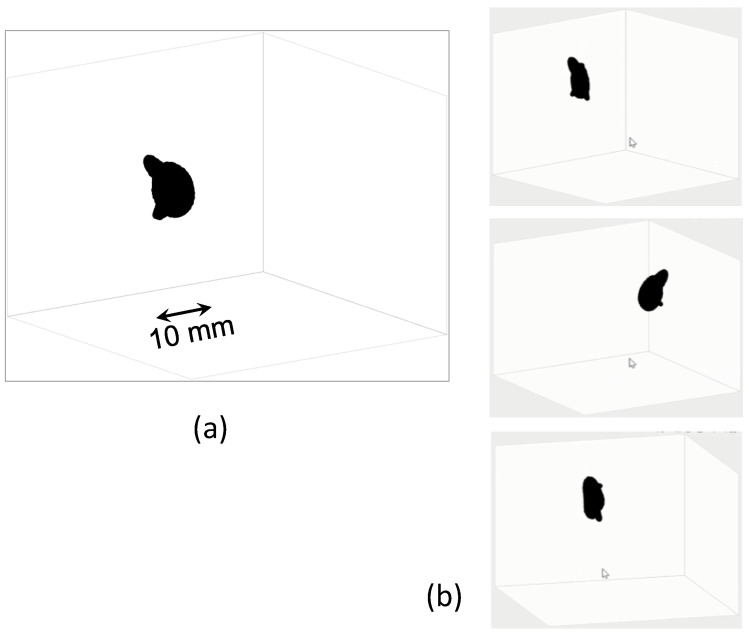
A 3D image of a volumetric absorber reconstructed from a single blurred 2D transillumination image: (**a**) one view angle, and (**b**) multiple view angles.

Figure 17 portrays the result of 3D reconstruction as viewed from different view angles. Even from the single blurred 2D image as in Figure 16, we were able to reconstruct the clear 3D structure of the absorber in the tissue-simulating turbid medium.

## 5. Conclusions

To visualize the internal light-absorbing structure of an animal body, NIR-TI is a promising technique. Nevertheless, it has not been widely used in biomedical fields. Two major difficulties have arisen to hinder the wider spread of this method. One is the severe blur caused by strong light scattering in animal tissues. Only the absorber structure close to the body surface can be seen. Another difficulty is the limitation of the 2D image. Without depth information, the usefulness of this method is greatly limited. In NIR-TI, we were able to obtain only the blurred 2D surface or subsurface information. To resolve these difficulties, we developed two techniques to reconstruct a clear 3D image of the absorbing structure in a turbid medium from a single blurred 2D transillumination image.

In the first technique, 1D information of the image is used. We calculated many intensity profiles across a curvilinear cylindrical absorber in various conditions such as different absorber diameters and different depths in a turbid medium. Then, they were included in a database of the intensity profiles. In the blurred transillumination image, we obtained the intensity profile across the linear absorber. We measured the contrast and the spread of the profile. Using the combination of these two parameters, we found the closest case in the database which provides the absorber diameter and depth. In simulation, one-to-one correspondence between these parameters with no cross-talk was newly found in the practical measurement range. The feasibility of the proposed technique was verified by experimentation. The submillimeter spatial resolution was confirmed with an average error of micrometer order. Because this technique is simple and fast, it is suitable for implementation in real-time diagnostic devices such as devices to visualize the 3D blood vessel network.

For the second technique, 2D information of the image is used. The original blurred transillumination image is deconvolved with the depth-dependent PSF. By changing the depth, we can obtain multiple deconvolved images. In each deconvolved image, we find the in-focus part. By selecting the best-focus part among the multiple deconvolved images, we can obtain an all-in-focus image with the depth distribution over the whole image. The difficulty of image noise caused by deconvolution was solved using the noise-robust selective fusion method. This report describes the first attempt to use this method for transillumination images. Depth estimation accuracy was evaluated by experimentation. High linear correlation (*R*^2^ > 0.99) between the given and estimated depths in the depth range of 0–13.5 mm was confirmed. The estimation errors were less than 15% and 7.4% before and after the linear calibration, respectively. The feasibility of the proposed technique was verified using 3D reconstruction of a volumetric absorber with irregular shape. This technique is applicable to visualize a light-absorbing object in a turbid medium such as a cancerous lesion in normal body tissues. Both techniques are straightforward and require neither mechanical scanning nor great amounts of computational power. Based on these merits, their potential for medical use is promising.

In transillumination imaging, the background non-uniformity degrades the image of the major structure of interest. The background removal technique used in this paper in not always effective with a living animal body. Application of the proposed technique to an actual animal body image is left as a task for future research.
